# From external rewards to intrinsic drive: A self-determination theory analysis of paramedic motivations for postgraduate education

**DOI:** 10.4102/hsag.v31i0.3198

**Published:** 2026-02-03

**Authors:** Naseef Abdullah, Patricia McInerney, Simpiwe Sobuwa

**Affiliations:** 1 Emergency Medical Services, Western Cape Government Health and Wellness, Cape Town, South Africa; 2 Department of Emergency Medical Care & Rescue, Faculty of Health Sciences, Durban University of Technology, Durban, South Africa; 3 Research Office, Faculty of Health Sciences, University of the Witwatersrand, Johannesburg, South Africa; 4 Department of Emergency Medical Sciences, Faculty of Health and Wellness Sciences, Cape Peninsula University of Technology, Cape Town, South Africa

**Keywords:** postgraduate education, paramedics, self-determination theory, motivation, qualitative research, professional development

## Abstract

**Background:**

Paramedicine research is expanding, yet non-paramedic researchers dominate knowledge production. This gap requires increased paramedic participation in postgraduate education to develop independent scholars and enhance discipline-specific research capacity.

**Aim:**

To examine paramedics’ motivations for postgraduate education using self-determination theory (SDT).

**Setting:**

The study utilised virtual platforms to engage South African-trained emergency care practitioners practising globally.

**Methods:**

This qualitative study explored motivations among 51 paramedics from operational, academic, and management sectors in South Africa and internationally. Semi-structured interviews and focus groups were analyses using SDT as a framework.

**Results:**

The study revealed that paramedics’ motivations for pursuing postgraduate education varied along a spectrum of regulatory styles, ranging from external regulation to intrinsic motivation. External regulation was driven by financial incentives and career advancement opportunities. Identified regulations reflected personal recognition of education’s importance and qualification value. Integrated regulations demonstrated an alignment between education pursuits and personal beliefs or life goals. Intrinsic motivation was characterised by desires for professional growth and development.

**Conclusion:**

The heterogeneous nature of paramedics’ postgraduate education motivation has significant policy and institutional implications. Traditional one-size-fits-all approaches may be ineffective.

**Contribution:**

Educational institutions and professional development programmes should align initiatives with individual motivational profiles, emphasising autonomy-supportive environments and connecting learning opportunities to personal values and professional aspirations to optimise engagement and outcomes.

## Introduction

Paramedics play a critical role in the healthcare system as they are often the first point of contact in emergencies (Williams, Beovich & Olaussen 2021). Time-sensitive emergency care and rapid transportation to the nearest appropriate medical facility can be the difference between life and death. Paramedicine is an expanding profession in many countries, transitioning from primarily vocational training to a more academic focus in universities (Beovich, Olaussen & Williams 2021). An important step that has aided this progress and must continue, is the growth in research (Beovich et al. 2021; Dippenaar & Marr 2021). The annual publication rate of paramedicine-related publications increased two-fold between 2010 and 2019, indicating significant growth in paramedicine research (Beovich et al. 2021). However, 70% of the most-cited authors were from a single institution in Australia, and only one of the top 10 cited authors was a paramedic (Beovich et al. 2021). The emerging research ecosystem in South Africa demonstrates considerable promise, with a recent editorial commentary identifying ‘a surge of developments in this field and in the research being generated, particularly from South Africa’, highlighting the country’s emerging role as a regional research hub for prehospital emergency care in Africa (Hodkinson 2024). Despite this surge, the field remains dominated by authors from other professions who are the key drivers of prehospital research, highlighting a critical gap in practitioner–researcher representation. Thus, there is a need for more paramedics to pursue postgraduate education and become independent scholars to increase prehospital knowledge production.

However, these practitioners have jobs, families and other responsibilities that conflict with their opportunities to pursue postgraduate education. Moreover, there is no increased scope of practice or specialisation for pursuing postgraduate education for South African paramedics. This presents a need to investigate the motivation of paramedics pursuing postgraduate education to increase the profession-specific body of knowledge (Olaussen, Beovich & Williams 2021). Motivation theories can aid in identifying factors and guiding strategies to promote postgraduate education in paramedicine. Deci and Ryan’s self-determination theory (SDT) is one such theory that claims that people have three intrinsic psychological demands that are the foundation for complete psychology: competence, relatedness and autonomy (Jansen in de Wal et al. 2020). These needs are necessary for a person’s development and psychological well-being (Luhanga et al. 2022). These requirements are not learned; they are an innate part of human nature, contributing to optimal functioning and well-being (Luhanga et al. 2022). Furthermore, they are viewed as vital in understanding the underlying motivations for people’s pursuit of goals (Ryan & Deci 2000).

Self-determination theory defines three needs (competence, relatedness and autonomy) (Ganotice et al. 2021). The quest for competence is the intrinsic desire to gain expertise and effectively handle present tasks. Relatedness is the desire to connect, communicate and build compassion for others. Relatedness focuses on the sensation of inclusion (Tsoi et al. 2016a). Autonomy refers to the desire to control one’s life and live according to one’s principles and ideas (Tsoi et al. 2016a). However, being independent does not imply being cut off from others; instead, it entails actively exercising purposeful control over one’s life (Deci & Ryan 2000; Ryan & Deci 2000). According to SDT, motivation has two dimensions: intrinsic and extrinsic (Ryan & Deci 2000).

Intrinsic motivation is the most ideal because it is entirely self-directed and has been linked to various positive outcomes such as effective metacognitive strategies, increased determination, a stronger intention to persist, and improved perseverance (McAnally & Hagger 2024). Furthermore, intrinsic motivation has been found to provide positive academic results and enhance comprehension of the learning topics beyond what would be expected (Grassinger et al. 2024). Nonetheless, SDT holds that increasing levels of desire do not always result in more desirable outcomes, as other factors may impact the outcomes. For example, a student with intrinsic drive may have difficulties pursuing postgraduate education because of personal challenges.

External regulation is the most basic extrinsic motivation in which external demands influence individuals’ behaviour. People engage in specific behaviours to avoid a potential punishment or to obtain a tangible incentive. Introjected regulation refers to those who engage in activities under external pressure to avoid guilt or boost their pride or ego. External regulation influences behaviour by imposing external consequences, whereas introjected regulation includes individuals delivering contingent consequences to control their behaviour (Deci & Ryan 2000; Ryan & Deci 2000).

Identified regulation is a form of extrinsic motivation in which an individual recognises the personal importance of an activity and takes ownership of it. Integrated regulation is the ultimate level of extrinsic motivation in which a person truly understands their actions by reflecting on their ideas and desires. Integrated regulation has some parallels to intrinsic motivation. Nevertheless, it varies because the motives for engaging in the conduct are external to the individual rather than derived from innate satisfaction (Deci & Ryan 2000; Ryan & Deci 2000).

Doctoral candidates driven by more autonomous forms of motivation (intrinsic, integrated and identified) tend to demonstrate increased performance, well-being and persistence than those driven by controlled forms of motivation – introjected and external (Litalien et al. 2024). Furthermore, several predictors such as the satisfaction of fundamental psychological needs (autonomy, competence and relatedness) and social capital may affect these motivating characteristics (Litalien et al. 2024). Understanding these dynamics may help identify postgraduate students with reduced performance and persistence, enabling focused strategies to increase autonomous motivation.

The study aimed to identify paramedics’ motivations for pursuing postgraduate education in paramedicine. Self-determination theory was used as a framework to analyse the findings. Some studies have used SDT to examine self-reported motivation in education (Litalien et al. 2024; Messineo, Allegra & Seta 2019; Sobuwa & Lord 2019). However, no studies have examined paramedics’ motivations to pursue postgraduate studies.

## Research methods and design

### Study design

This was a qualitative study guided by a social realist paradigm, which assumes that while individual experiences and meanings are socially constructed, they are shaped by real underlying structures such as professional hierarchies, educational systems, and psychological needs. This ontological and epistemological stance supports the use of SDT as a framework for interpreting motivation as both socially influenced and individually experienced.

### Participants and recruitment

At the time of the study, 851 paramedics on the emergency care practitioner (ECP) register were registered with the Health Professions Council of South Africa (HPCSA). Registration with the HPCSA is mandatory for any healthcare professional treating patients in South Africa. Emergency care practitioners hold either a 4-year degree in emergency medical care or a 3-year diploma, followed by a 2-year part-time degree. They represent the highest qualification level for emergency care personnel in the South African prehospital context and are equipped with skills such as rapid sequence intubation and thrombolysis.

The sample included ECPs who practised their profession abroad and in South Africa. While all participants were registered as ECPs in South Africa, many work abroad in the Middle East because of high remuneration opportunities (Tiwari et al. 2021). The participants were recruited via purposive and snowball sampling techniques and were drawn from three prehospital groups: operational, academic and management. The academic group included undergraduate and postgraduate course conveners, the operational group working in direct patient care, and the management group within South Africa’s emergency medical services. Practitioners not registered as ECPs were excluded from the study.

### Data collection

The semi-structured interviews and focus group discussion guides were developed based on the study’s objectives and a prior literature review. These guides were refined in collaboration with all the authors and piloted with South African emergency care technician (ECT) graduates who had elected to seek or forego pursuit for the ECP qualifications. Emergency care technicians are mid-level prehospital care practitioners with a 2-year National Certificate in Emergency Care. Following the pilot study, no amendments were necessary. The first author conducted all the interviews in English.

A research advertisement was disseminated across various prehospital-specific social media platforms, EMS-specific training institutions, and related postgraduate support platforms and organisations. These endpoints and individuals were asked to forward the research advertisement to potential participants. Of the 71 people who expressed interest in participating in focus group discussions, 51 were ultimately recruited for the study.

Given the coronavirus disease 2019 (COVID-19) pandemic and participants’ diverse geographical locations, online platforms were used for engagement. These sessions were hosted on Microsoft Teams between 31 May 2022 and 30 June 2022. The participants received a study information letter and an expression of interest form before sessions, facilitating their categorisation into operational, academic and managerial groups. Informed consent to audio recording was obtained.

The authors conducted semi-structured interviews (*n* = 2) and focus group discussions (*n* = 6) with ECPs (*n* = 33), facilitators in undergraduate and postgraduate programmes (*n* = 15), and managers in the EMS profession (*n* = 3). To address potential hierarchical power dynamics that could inhibit open discussion during focus groups, two senior management participants (the Director of EMS and one senior manager) were interviewed individually rather than included in the focus groups. All the participants provided informed written consent. All interviews and focus groups were audio-recorded and lasted between 60 min and 80 min. These recordings were transcribed verbatim and anonymised for analysis.

### Data analysis

Data analysis followed a theory-informed, deductive approach using SDT as the guiding analytical framework. Given the study’s focus on exploring paramedics’ motivations for pursuing postgraduate education, SDT offered a structured and conceptually robust foundation. The first author, who is also an experienced ECP, conducted all coding and analysis. This allowed for close engagement with the data and sensitivity to contextual nuances within the professional paramedic environment.

A structured coding framework was developed in advance based on SDT’s five regulatory styles: external, introjected, identified, integrated and intrinsic. Each style was defined and operationalised based on the SDT literature and adapted to reflect the language and context of paramedicine. Transcripts were uploaded into NVivo 12.6.1 software, where the first author conducted iterative readings during the familiarisation phase. Coding was applied systematically to identify expressions of motivation that aligned with the SDT regulatory continuum. Participant accounts that offered particularly rich insights into motivational processes were flagged as exemplar cases for deeper interpretive analysis.

To enhance trustworthiness and interpretive credibility, the second author, with expertise in qualitative methods, reviewed a sample of the coded transcripts. This process enabled critical reflection on the consistency and theoretical alignment of the coding. Rather than seeking inter-coder reliability in a quantitative sense, the review focused on ensuring conceptual coherence and refining code definitions through dialogue. Analytical discussions between the two researchers supported the iterative refinement of the coding framework and resolved interpretive discrepancies with reference to SDT scholarship.

The coded data were organised by regulatory style to enable pattern recognition across participants and professional roles. Constant comparison techniques were employed to examine the range and complexity of motivational experiences within and across categories. Illustrative excerpts were selected to highlight typical and divergent examples of each motivational style. As themes solidified, the framework was refined to reflect the nuances of the data while maintaining theoretical congruence with SDT.

### Trustworthiness of the study

The study was conducted within a social realist paradigm, which recognises that individuals’ experiences and motivations are shaped by both subjective meaning-making and objective structural conditions. This philosophical position acknowledges that while participants construct their own realities, these constructions are influenced by real-world factors such as institutional expectations, professional norms, and educational structures.

Accordingly, the study applied strategies that ensured methodological congruence with this paradigm, drawing on Guba’s criteria for trustworthiness: credibility, transferability, dependability, and confirmability. To enhance credibility, the authors included a diverse sample of ECPs from different professional roles and geographic contexts, allowing for a richer understanding of motivational experiences across settings. Focus group discussions facilitated interaction among peers, encouraging open and honest dialogue, while member checking allowed participants to verify the accuracy of their transcribed accounts.

Transferability was supported by providing detailed contextual information about the research setting, participant characteristics, and sampling approach, allowing readers to assess the applicability of the findings to their own contexts. Dependability was addressed through a clearly documented analytical process, including an audit trail of coding decisions and team discussions to refine the coding framework. Confirmability was supported by collaborative review of coded transcripts and ongoing discussion of interpretations to ensure alignment with the SDT framework and the study’s social realist orientation. The study’s use of a theory-informed analytical framework (SDT), combined with reflexive and transparent practices, ensured that interpretations were grounded in both the participants’ lived realities and the structural contexts within which they operate, reflecting the core tenets of social realism.

### Researcher positionality

The first author was an active ECP in the United Arab Emirates during data collection. His lived experience as an ECP provided an authentic perspective on the research problem. This was seen as a strength rather than a risk in contributing to and comprehending the study’s findings. Furthermore, the other two writers oversaw the data gathering and analysis.

### Ethical considerations

The data were de-identified to protect anonymity during analysis and reporting. Pseudonyms were used in the study (note Table 1). Furthermore, all the participants were informed of their ability to freely consent to participate in the study with no repercussions (autonomous individuals). While confidentiality in focus group discussions cannot be assured, members were encouraged to avoid discussing the conversations with other parties after the session. All the data points were saved on a password-protected device. The Durban University of Technology Institutional Research Ethics Committee (IREC 089/21) approved this study.

**TABLE 1:** Motivation for pursuing postgraduate education.

Motivation	Regulatory style	Comments	Rationale
Extrinsic	External regulation	‘I can do my PhD for free because I am a university staff member; means that it doesn’t cost me an extra R25000† every year.’ (Steve)	The emphasis is on an external benefit (free education) or avoiding a cost (not having to pay R25 000). The motivation appears to be mostly dependent on the external element rather than personal interest or value in the PhD itself.
Extrinsic	Identified	‘When the opportunity to work abroad presented itself, I took it, I grabbed it with both hands. It has provided me with the opportunity to get my Master’s while being financially stable.’ (Richard)	Richard deliberately opted to seize the opportunity (‘I took it, I grabbed it with both hands’), expressing personal support for the action. They recognise the importance of the opportunity for their own development (obtaining a Master’s degree). The decision appears to be consistent with their personal goals and ideals.
Extrinsic	Identified	‘If I’m gonna [*going to*] sit as the head of an institution or a head of a service, I need to be able to deliver, and if I am gonna [*going to*] be able to do that, I need to capacitate myself and the only way to do that is to go [*and*] get educated.’ (Sizwe)	Sizwe is pursuing further studies (a PhD) not because he enjoys studying (although he may), but because he understands its relevance in reaching his goal of being a capable leader in his area. The statement emphasises the instrumental role of education in obtaining a desired outcome, which is a crucial feature of identified regulation.
Extrinsic	Identified	Education is power chief‡ … ‘If I introduce myself as doctor, then I get the attention, but if I say I’m a paramedic, then I’m just seen as an ambulance driver … We have to go get those qualifications to be able to stand up and make ourselves heard, to change this narrative.’ (Eric)	Eric perceived a definite benefit in receiving attention, being heard and changing the narrative. He actively recognised the value of education and qualifications in achieving his aims. Motivation is more internalised than external regulation, but it is still not entirely integrated into his essential values or identities.
Extrinsic	Integration	‘We are working abroad, and that makes us decision makers and actually enables us to go out and seek further development.’ (Zina)	Zina mentions that working overseas allows them to be decision-makers and pursue further development. This demonstrates that their desire to work overseas is not only about external incentives (such as increased wages or a new experience) but also about intrinsic values like autonomy and personal improvement.
Extrinsic	Integration	‘I want to see myself reach those heights, whereby I am capacitated to the point where I can make the necessary change needed among our people. I need to hold those qualifications and be in the right seat, be capable of doing what is expected [*of me*] and also advance the narrative, because postgraduate [*education*] allows us to research what we believe in.’ (John)	John’s motivation for pursuing postgraduate study goes beyond its instrumental value, as they are driven by a profound sense of purpose and identity. There is an aspiration to utilise their education to effect positive change in their community, propel the discourse forward, and investigate topics aligned with their convictions. All of these are inherent values that are profoundly embedded in their identity.
Extrinsic	Integration	‘These qualifications allow and actually make us capable of making meaningful change in the workplace … the onus is upon you as the [*postgraduate*] qualification holder … you decide your purpose, you position yourself to make the relevance.’ (David)	The phrase ‘you decide your purpose, you position yourself’ conveys a strong sense of autonomy and self-determination. This extends beyond simply recognising the significance of the qualifications to fully incorporating them into one’s sense of self and life path.
Intrinsic	Intrinsic regulation	‘As a guy who’s always wanted to seek growth and development, and that’s the reason why I find myself time and time really pushing up with the ladder … I want growth. I want to challenge myself, and I want to see how far I can take this thing.’ (Sizwe)	Sizwe’s drive for both professional and personal development seems to come from a sincere desire for professional development and an innate appreciation of the growth process. This has less to do with receiving praise from others, avoiding penalties, or even being aware of one’s own significance than it does with the inner fulfilment that comes from pushing oneself to the limit and realising one’s potential.

† , US$1435 using the exchange rate on 23 September 2024.

‡ , Colloquial term often used when addressing strangers in South Africa.

## Results

Table 1 presents illustrative participant quotes mapped onto the regulatory styles of SDT, together with explanatory notes on how each account was classified. The table is not intended as a comprehensive list of all responses but rather as exemplar cases that highlight the range of motivational drivers identified in this study. These examples serve as anchors for the interpretive narrative that follows, demonstrating how participants’ motivations varied along the continuum from external to intrinsic regulation.

**FIGURE 1:**
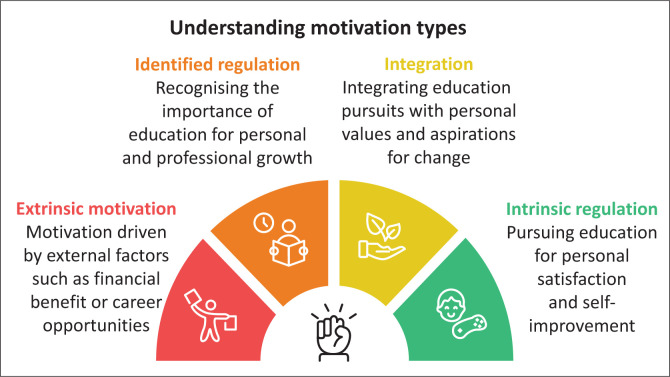
Abstract infographic.

Analysis of participants’ accounts revealed a spectrum of motivational styles ranging from externally driven to deeply internalised and intrinsic (see Figure 1). While the regulatory categories of SDT provided a useful framework, participants’ narratives highlighted the fluid and overlapping nature of these motivations.

External regulation was evident where postgraduate study was pursued primarily for material benefit or to avoid financial burden. For instance, some participants framed opportunities such as staff fee exemptions as the main incentive to register for postgraduate education. These accounts placed little emphasis on the personal or professional meaning of education, underscoring how structural enablers and constraints can act as strong motivators.

Moving along the continuum, identified regulation was the most prevalent form of motivation. Participants frequently recognised the instrumental value of qualifications in achieving career mobility, leadership positions or international opportunities. For example, some described postgraduate degrees as essential for securing credibility in senior roles or for changing how paramedics are perceived within the broader healthcare community. These accounts reflect a process of recognising the importance of education for personal and professional advancement, even when the immediate motivation was not the enjoyment of postgraduate education itself.

Integrated regulation appeared when participants described postgraduate education as aligned with their deeper values, identities and aspirations. Unlike identified regulation, these accounts went beyond instrumental reasoning to articulate postgraduate education as part of a broader life purpose, such as making meaningful change in communities, contributing to the profession’s development or embodying the values of leadership. Such examples illustrate how postgraduate education was internalised into participants’ sense of self, reflecting the transformative role of education in shaping both professional and personal identities.

Intrinsic motivation was rare in the dataset, with only one participant describing postgraduate education as inherently fulfilling. This individual emphasised curiosity, personal challenge and the enjoyment of intellectual growth. While uncommon, this account illustrates that for at least some paramedics, the pursuit of education can be driven by an internal desire for growth rather than external or instrumental factors.

## Discussion

Motivation in healthcare education has been studied in other disciplines such as nursing and medicine (Osborne, Anderson & Robson 2025; Sušilović et al. 2025). However, research exploring why paramedics pursue further education remains limited. This study contributes to this gap by exploring the motivations of paramedics pursuing postgraduate education using SDT as a framework. The study’s findings revealed a spectrum of regulatory styles ranging from external regulation to intrinsic motivation, with identified and integrated regulation being the most common. This suggests that many paramedics have internalised the value of postgraduate study, aligning education with personal and professional goals.

At one end of the spectrum, external regulation was evident in cases where financial incentives drove engagement, consistent with previous research showing that financial incentives can play a significant role in motivating healthcare professionals to pursue further education (Tsoi et al. 2016b). In the paramedicine context, external regulation is also reinforced by systematic factors such as career progression requirements in academia and institutional incentives like tuition subsidies (Abdullah, Sobuwa & McInerney 2024). Identified regulation was another dominant finding in this study, which was demonstrated through recognition of postgraduate education as essential for leadership or credibility. This finding is consistent with studies in other healthcare professions, such as nursing, where motivations related to job opportunities and career security are significant factors influencing students’ decisions (Messineo et al. 2019). Integrated regulation, which aligns education with identity and purpose, was strongly represented in the current study’s data. This resonates with studies on PhD motivation that highlight the role of integrated forms of regulation in sustaining persistence and well-being (Litalien et al. 2024). Finally, although intrinsic motivation was rare, its presence is consistent with SDT’s assertion that autonomous forms of motivation, even when less common, can be highly beneficial for learning outcomes (Grassinger et al. 2024).

The prevalence of identified and integrated regulation in this study’s findings is particularly noteworthy. This suggests that many paramedics have internalised the value of postgraduate education, seeing it as being aligned with their personal goals and professional identity. This contrasts somewhat with studies in other healthcare fields, where external regulation has often been found to be more dominant (Tsoi et al. 2016a).

### Implications for education

The diverse motivational factors identified in this study have significant implications for educational institutions seeking to develop supportive settings that foster intrinsic motivation, autonomy and professional purpose among paramedic students. Educational institutions should consider developing specialised postgraduate programmes tailored to distinct paramedic career pathways, including prehospital critical care, education, management, and rescue operations. Such targeted offerings would better align with professional interests and career trajectories while addressing the operational, academic and management competencies required within contemporary paramedicine. Furthermore, institutions must collaborate with employers and professional regulatory bodies to ensure postgraduate qualifications receive appropriate recognition and translation into tangible career advancement opportunities. This alignment between educational achievement and professional progression is essential for sustaining motivation and validating educational investment.

### Limitations

Several limitations should be acknowledged when interpreting these findings. While this study included paramedics from different professional groups (operational, academic and management), the findings may not be generalisable to the broader population of paramedics and healthcare practitioners in similar low- and middle-income countries (LMICs) context. The study specifically focused on postgraduate education and not on motivations for broader professional development. Although participants practised in different parts of the world, they were all trained in South Africa. The homogeneity of educational background within the South African context, while providing internal consistency, limits the external validity of results to other LMIC settings with different training paradigms. Future research should investigate the impact of regulatory frameworks on the motivations for postgraduate education among diverse healthcare providers within the South African healthcare system, as well as comparative studies examining professional development motivations across different LMIC contexts with varying educational and regulatory structures.

## Conclusion

The study highlights the complexities of human motivation in the context of postgraduate education and career advancement in paramedicine. It emphasises the importance of nuanced approaches that recognise and cater to various motivational styles. This could lead to more effective strategies for encouraging and supporting lifelong learning and professional development.
